# Perinatal Changes of Cardiac Troponin-I in Normal and Intrauterine Growth-Restricted Pregnancies

**DOI:** 10.1155/2007/53921

**Published:** 2007-07-01

**Authors:** Nicoletta Iacovidou, Maria Boutsikou, Demetrios Gourgiotis, Despina D. Briana, Stavroula Baka, Venetia-Maria Vraila, Louiza Kontara, Demetrios Hassiakos, Ariadne Malamitsi-Puchner

**Affiliations:** ^1^Neonatal Division, 2nd Department of Obstetrics and Gynecology, Athens University Medical School, Athens 10682, Greece; ^2^Research Laboratories, 2nd Department of Paediatrics, Athens University Medical School, Athens 10682, Greece

## Abstract

Intrauterine growth restriction (IUGR) implies fetal hypoxia, resulting in blood flow redistribution and sparing of vital organs (brain, heart). Serum cardiac Troponin-I (cTnI), a well-established marker of myocardial ischaemia, was measured in 40 mothers prior to delivery, the doubly clamped umbilical cords (representing fetal state), and their 20 IUGR and 20 appropriate-forgestational-age (AGA) neonates on day 1 and 4 postpartum. At all time points, no differences in cTnI levels were observed between the AGA and IUGR groups. Strong positive correlations were documented between maternal and fetal/neonatal values (*r* ≥ .498, *P* ≤ .025 in all cases in the AGA and *r* ≥ .615, *P* ≤ .009 in all cases in the IUGR group). These results may indicate (a) normal heart function, due to heart sparing, in the IUGR group (b) potential crossing of the placental barrier by cTnI in both groups.

## 1. INTRODUCTION

Cardiac troponin (cTn), an inhibitory protein complex located on the actin filament in all striated muscles, consists of three subunits T, I, and C [1] and coordinates striated muscle contraction in response to voltage changes [2]. cTnI is encoded by specific genes [3], blocks the formation of actin-myosin bridges [4] and since it is not found in skeletal muscles [5, 6], it is considered a highly specific indicator of myocardial injury in adults [4]. However, studies have shown that cTnI is also successfully used in the diagnosis of myocardial injury in neonates with asphyxia, respiratory distress syndrome, and septic or cardiogenic shock [7]. Although the half-life of cTnI is relatively short (90 minutes), its diagnostic time range is unusually wide (ranging from a few hours to 10–14 days after the episode of myocardial injury) as a consequence of intracellular compartmentation [7]. 

Intrauterine growth restriction (IUGR) caused by the chronic malnutrition and hypoxia [8, 9] (consequent to deficient placental transport of nutrients and oxygen [10], asymmetrical pattern of IUGR) is characterized by blood flow redistribution to vital organs (brain, myocardium, and adrenal glands), while other organs are deprived from sufficient blood flow. This phenomenon called “the brain-sparing effect” is usually accompanied by oligohydramnios [11, 12].

Taking into account the brain-sparing effect, this study was based on the hypothesis that circulating cTnI levels should not differ between IUGR and appropriate-for-gestational age (AGA) full-term infants. Therefore, we aimed to determine circulating cTnI levels in IUGR and AGA pregnancies at time-points characteristic for intra-and extrauterine life, and correlate determined levels with gestational age, gender, and mode of delivery.

## 2. SUBJECTS AND METHODS

The Ethics Committee of our teaching hospital approved the study protocol. All included mothers provided signed informed consent before recruitment. Forty parturients giving consecutively birth either to 20 AGA or 20 asymmetric IUGR full-term singleton infants with a birth weight below the 3rd customized centile were included in the study. The Gestation Related Optimal Weight computer-generated program [13, 14] was used to calculate the customized centile for each pregnancy, taking into consideration significant determinants of birth weight, as maternal height and booking weight, ethnic group, parity, gestational age, and gender [13]. Causes of IUGR were identified in each one of our 20 IUGR cases. Thus, nine mothers were presented with preeclampsia and the remaining 11 were presented with gestational hypertension in addition to other pathological conditions, such as iron-deficient anemia (3 cases), gestational diabetes mellitus (2 cases), hypothyroidism (3 cases), extreme obesity (2 cases), and cardiac arrhythmias (1 case). Five of the above 11 women were smoking more than 10 cigarettes per day during the whole duration of pregnancy. 

Doppler studies were performed in the IUGR group every 10–15 days, starting from the 32nd gestational week. During each Doppler velocimetry evaluation, three consecutive measurements of the pulsatility index (PI) of the studied vessel were done and the mean value was recorded. Concerning uterine and umbilical arteries [8, 15], mean PI values were progressively found to be in the upper physiological limits for the corresponding gestational age in 13 cases (ranging between the 90th and the 95th percentiles), while in the remaining seven cases, PI values showed increased impedance to flow, being above the 95th percentile for gestational age. Regarding middle cerebral arteries [16], Doppler studies showed resistance to be in the lower physiological limits for gestational age, indicating the initiation of blood flow redistribution process. Nevertheless, amniotic fluid was diminished in all IUGR cases. For the evaluation of the amniotic fluid, the largest fluid column on the vertical plane was assessed and was defined as diminished, if <2 cm. Furthermore, placental weight was reduced ranging from 255 to 400 g.

In contrast, in the AGA group, mothers were healthy and were either nonsmokers or had abstained from smoking during pregnancy. Moreover, placentas were normal in appearance and weight.

All neonates had no symptoms of intrauterine infection or stigmata of genetic syndromes. One- and five-minute Apgar scores were ≥8 and umbilical cord pH values were ≥7.25 in all IUGR cases and AGA controls. The demographic data of participating infants and their mothers are shown in Table 1. 

Blood was collected at specific time points as follows: (1) from the mother (MS) during the first stage of labour, or before receiving anaesthesia in cases of elective caesarean section, (2) from the doubly clamped umbilical cord (UC), reflecting the fetal state, and (3) from the neonates on postpartum days 1 (N1) and 4 (N4), which are characteristic for transition and stabilization to extrauterine life, respectively. Blood was collected in pyrogen-free tubes and was immediately centrifuged after clotting. The supernatant serum was stored at −80°C until assay. cTnI was determined by a commercially available enzyme immunoassay kit [troponin I (human cardiac specific) enzyme immunoassay test kit, catalogue number 1105Z, Diagnostic Automation Inc, Calabasas, Calif, USA]. All specimens were run in duplicate. The detection limit was 0.1 ng/mL. The intra- and interassay coefficients of variation were 2.8% and 6.7%, respectively. The antibodies used in this assay were highly specific for cTnI and there was no cross-reactivity with cTnT or skeletal troponin-T. 

## 3. STATISTICAL ANALYSIS

cTnI was normally distributed; thus, Anova for repeated measures, paired samples *t* test with Bonferroni correction for multiple comparisons, and Spearman's or Pearson's correlation coefficient were used as appropriate. *P* < .05 was considered statistically significant.

## 4. RESULTS

**Figure 1:**
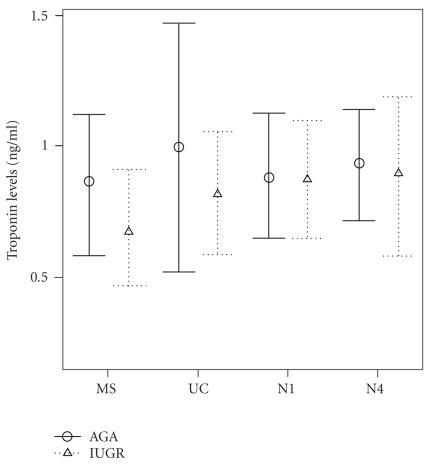
Cardiac troponin-I (cTnI) levels in maternal (MS), fetal (UC), neonatal day 1 (N1), and neonatal day 4 (N4) serum of intrauterine growth-restricted (IUGR) and appropriate-for-gestational age (AGA) groups. Error bars represent mean and 95% confidence interval of the mean.

Mean (SD) circulating cTnI levels in AGA and IUGR groups at all time points (MS, UC, N1, and N4) are shown in Figure 1. For all time points, no statistically significant differences in cTnI levels between IUGR cases and AGA controls were documented. 

In the AGA group, no statistically significant differences in cTnI levels were observed between MS, UC, N1, and N4. MS cTnI levels positively correlated with respective levels in UC, N1, and N4 (*r* = .536, *P* = .018, *r* = .498, *P* = .025 and *r* = .730, *P* < .001, resp.). Additionally, N4 cTnI levels positively correlated with UC and N1 levels (*r* = .492, *P* < .032 and *r* = .665, *P* = .001). As expected, birth weight positively correlated with gestational age (*r* = .562, *P* = .010).

In the IUGR group, MS cTnI levels were significantly lower compared to respective levels in N1 (*P* < .012) and N4 (*P* = .026). Additionally, MS cTnI levels positively correlated with respective levels in UC, N1, and N4 (*r* = .615, *P* = .009, *r* = .847, *P* < .001 and *r* = .770, *P* < .001, resp.). N1 cTnI levels positively correlated with N4 ones (*r* = .856, *P* < .001). Finally, gestational age positively correlated with birth weight (*r* = .555, *P* = .009). 

**Figure 2:**
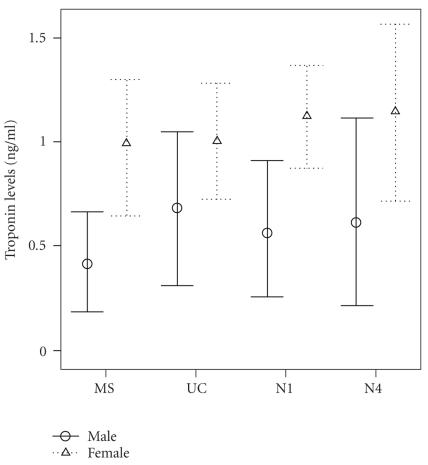
Cardiac troponin-I (cTnI) levels in maternal (MS), fetal (UC), neonatal day 1 (N1), and neonatal day 4 (N4) serum of pregnancies with intrauterine growth-restricted (IUGR) male and female offsprings.

The effect of gender on cTnI levels was found to be significant in the IUGR group (see Figure 2). Thus, cTnI levels were significantly lower in mothers with male offspring than female [regression coefficient b: −0.468, 95% CI: (−0.928/−0.008), *P* = .047]. Similarly, N1 cTnI levels were significantly lower in males than females [regression coefficient b: −0.487, 95% CI: (−0.948/−0.026), *P* = .04]. Lastly, gestational age and mode of delivery did not affect cTnI levels in both groups.

## 5. DISCUSSION

Although cTnI has been extensively investigated as a diagnostic and prognostic marker of myocardial damage and outcome in paediatric and adult population [17–19], data concerning neonates are relatively limited. In this respect, cTnI was increased in asphyxiated neonates compared to healthy ones, suggesting myocardial injury [1]. In addition, cTnI has been reported to be significantly elevated in the cord blood of critically ill newborns and even higher in nonsurvivors [20], implying that cTnI could serve as a predictor of mortality in this group of newborns. Furthermore, increased levels of cTnI were associated with a lower umbilical artery pH [21], and have been reported in sick preterm neonates with idiopathic respiratory distress [22]. 

Although some investigators claim that cTnI levels are undetectable in healthy adults and healthy pregnant women [23, 24], others have given reference ranges for this substance [25, 26]. McDonough et al. [27] suggest that under conditions of myocardial stress, ischaemia without myocyte necrosis may occur, resulting in intracellular degradation of troponin and release of its fragments, which are detected in the serum. The above explanation may justify the presence of troponin in the maternal circulation in this study.

On the other hand, it has been reported that healthy term newborns were presented with higher upper reference limits of circulating cTnI than adults [25, 26, 28]. No plausible explanation why this happens has yet been reached. Nevertheless, this fact may reflect programmed cell death, since apoptosis contributes to the neonatal adaptive changes in the heart postpartum [29, 30]. Otherwise, this fact could be attributed to different plasmatic eliminations of cTnI in neonates [31]. In accordance, our study showed that mothers demonstrated lower cTnI levels than their IUGR infants who had oligohydramnios and presumably impaired renal function. 

Araujo et al. [31] published reference values for cTnI in healthy term neonates, ranging between 0.01 ng/mL (2.5th centile) and 2.8 ng/mL (99th centile). The values of cTnI in both groups (IUGR and AGA) of the present study range within these limits; therefore, they suggest no evidence of myocardial damage (according to criteria for definition of myocardial injury [32]) and support our hypothesis that circulating cTnI levels should not differ between IUGR (below the 3rd customized centile) and AGA full-term infants, due to sparing of basic organs (e.g., brain, heart). In this respect, a previous study of ours reported that circulating levels of neurotrophins, which are important for pre- and postnatal brain development, did not differ between IUGR and AGA groups [33]. 

According to our results, cTnI seems to cross the placental barrier, since positive correlations between maternal and fetal/neonatal cTnI levels were documented for both IUGR and AGA groups. No relevant reports for cTnI exist in the literature; however, for cTnT experimental evidence suggests nontransplacental passage [34]. 

Our finding that maternal and neonatal cTnI levels are lower when the IUGR offspring is a male has not been previously reported. Relatively, Baum et al. [28] have demonstrated that male newborns have a significantly higher median cTnT value than females, while no respective difference was observed in their study concerning cTnI levels. On the contrary, other investigators give similar cTnT values for males and females [35]. 

In conclusion, circulating cTnI levels do not differ between IUGR cases and AGA controls, possibly due to the sparing of vital organs, like the heart. Furthermore, cTnI possibly crosses the placenta, as strong correlations between maternal and fetal/neonatal values are documented. Lastly, maternal and neonatal cTnI levels are lower when the IUGR offspring is of male gender. 

## Figures and Tables

**Table 1:** Demographic data for appropriate-for-gestational age (AGA) and intrauterine growth-restricted (IUGR) neonates and their mothers.

	AGA	IUGR	*P* value
	Mean (SD)	Mean (SD)		

Birth weight (g)	3356 (223)	2342 (229)	<.001
Birth weight customized centile	65.4 (12.6)	1.5 (1.5)	<.001
Gestational age (weeks)	38.4 (1)	38.1 (0.7)	NS
Gender			NS
Male	11 (55%)	11 (55%)	
Female	9 (45%)	9 (45%)	
Maternal age (years)	28 (4.0)	31 (5.0)	NS
Parity			NS
First	10 (50%)	11 (55%)	
Other	10 (50%)	9 (45%)	
Mode of delivery			<.013
Vaginal	13 (65%)	8 (40%)	
Caesarean section	7 (35%)	12 (60%)	
